# Bibliometric and visual analysis of research on the links between the gut microbiota and pain from 2002 to 2021

**DOI:** 10.3389/fmed.2022.975376

**Published:** 2022-11-15

**Authors:** Menghan Mao, Yanyu Zhou, Yingfu Jiao, Suqing Yin, Chiwai Cheung, Weifeng Yu, Po Gao, Liqun Yang

**Affiliations:** ^1^Department of Anesthesiology, Renji Hospital, Shanghai Jiao Tong University School of Medicine, Shanghai, China; ^2^Department of Anesthesiology, The University of Hong Kong, Hong Kong, Hong Kong SAR, China

**Keywords:** gut microbiota, pain, bibliometric analysis, hot spots, CiteSpace

## Abstract

**Background and aims:**

The gut microbiota is involved in the regulation of pain, which is proved by plenty of evidence. Although a substantial quantity of research on the link between the gut microbiota and pain has emerged, no study has focused on the bibliometric analysis of this topic. We aim to present a bibliometric review of publications over the past 20 years and predict research hot spots.

**Methods:**

Relevant publications between 2002 and 2021 were extracted from the Science Citation Index-Expanded (SCI-EXPANDED) of the Web of Science Core Collection (WoSCC) database on April 22, 2022. CiteSpace (version 5.8 R3c), VOSviewer, the Online Analysis Platform of Literature Metrology, and the R package bibliometrix were used to analyze and visualize.

**Results:**

A total of 233 articles have been published between 2002 and 2021. The number of publication outputs increased rapidly since 2016. The collaboration network revealed that the USA, Baylor College of Medicine, and Vassilia Theodorou were the most influential country, institute, and scholar, respectively. *Alimentary pharmacology and therapeutics* and *Gut* were the most co-cited journal and *Neurogastroenterology and Motility* was the most productive journal. Visceral sensitivity, fibromyalgia, gastrointestinal, chronic pain, stress, gut microbiome, LGG, brain-gut axis, SLAB51, and sequencing were the top 10 clusters in co-occurrence cluster analysis. Keyword burst detection indicated that the brain-gut axis and short-chain fatty acid were the current research hot spots.

**Conclusion:**

Research on the links between the gut microbiota and pain has increased rapidly since 2016. The current research focused on the brain-gut axis and short-chain fatty acid. Accordingly, the SCFAs-mediated mechanism of pain regulation will be a research direction of great importance on the links between the gut microbiota and pain. This study provided instructive assistance to direct future research efforts on the links between the gut microbiota and pain.

## Introduction

Pain is an unpleasant sensory and emotional experience associated with, or resembling that associated with, actual or potential tissue damage (1). The overall weighted age- and sex-standardized prevalence of pain across countries was approximately 27.5%, with a significant variation across countries (ranging from 9.9 to 50.3%) (2). Global burden of disease data indicates that chronic pain is a leading cause of years lived with disability, with painful conditions comprising 4 of 10 leading causes of disability in both developed and developing countries (3). It is estimated that one in three Americans suffer from chronic pain and cost between US$560 and US$635 billion per year in medical costs and lost productivity (4). In China, low back pain ranks as the second leading cause of years lived with disability burden disease (5). Up to 70% of patients with cancer suffer from pain (6). The age- and sex-adjusted prevalence of chronic regional pain (CRP) was 23.9% and chronic widespread pain (CWP) was 11.4% (7). However, the current treatment of pain is far from perfection. For example, the therapeutic alternatives for chronic pain are limited except for opioids. The widespread and improper use of opioids has resulted in overdose deaths and addictions in the USA (8). Therefore, the mechanism of pain is still needed to be further studied to develop new medications for pain management.

The gut microbiome comprises more than 100 species and 7,000 strains and is the most complex microecological system in our body (9). It mainly contains bacteria, archaea, fungi, protozoa, and viruses (10). The microbiota-gut-brain axis involves bidirectional communication between the gut microbiome and the brain, including the immune, endocrine, systemic, and neuronal pathways (11). An increasing body of evidence suggests that the perturbation of the microbiota-gut-brain axis is involved in central nervous systems (CNS) diseases, such as depression (12, 13), anxiety (14), Alzheimer’s disease (15, 16), autism (17, 18), and Parkinson’s disease (19, 20). The relationship between the gut microbiota and pain also received increasing attention. The microbiota-derived mediators include PAMPs, toxins, SCFAs, KYNA, cytokines, neurotransmitters (e.g., GABA and 5-HT), and so on, which have an impact on both the peripheral and CNS. On one hand, the gut microbiota directly or indirectly regulates the peripheral nervous system under chronic pain conditions. For example, microbiota-derived mediators can directly regulate the neuronal excitability of primary sensory neurons in DRGs through activation of TLRs, TRP, GABA receptors, and acid-sensing ion channels (21, 22), or indirectly regulate primary sensory neurons in DRGs through activation of non-neuron cells to release pro-inflammatory cytokines, anti-inflammatory cytokines, chemokines, and neuropeptides (23). On the other hand, the gut microbiota may regulate neuroinflammation-mediated central sensitization *via* the regulation of microglia by its metabolites SCFAs (19, 23). Research showed that both antibiotics (24) and probiotics (25) can alter visceral pain-related responses. Administration of *Lactobacillus acidophilus* induced μ-opioid receptors in rodents’ colonic epithelial cells and application of *Bifidobacteria* reversed CRD-induced visceral hypersensitivity in mice (26, 27). The gut microbiota promotes the development of chemotherapy-induced mechanical hyperalgesia, which was reduced in germ-free mice (28). Inflammatory pain was also reduced in GF mice compared with conventional mice (29). An elegant review by Guo et al. provides a comprehensive view of the role of gut microbiota in pain regulation and proposes that targeting the gut microbiota by diet or pharmabiotic intervention is a promising approach to the management of chronic pain (23). Although a substantial quantity of research on the links between the gut microbiota and pain has emerged, no study has focused on the bibliometric analysis of this topic.

Bibliometric analysis is a branch of informatics that quantitatively analyzes patterns in the scientific literature in order to understand emerging trends and the knowledge structure of a research field (30). This study aimed to present the first bibliometric analysis of research on the links between the gut microbiota and pain over the past 20 years based on the data from the Web of Science Core Collection (WoSCC) database. We used literature metrology characteristics to assess the research output, impact, collaboration, and identify hotspots on the gut microbiota research in the pain field and discussed the trends in this field over the next few years.

## Materials and methods

### Data sources and search strategies

**FIGURE 1:**
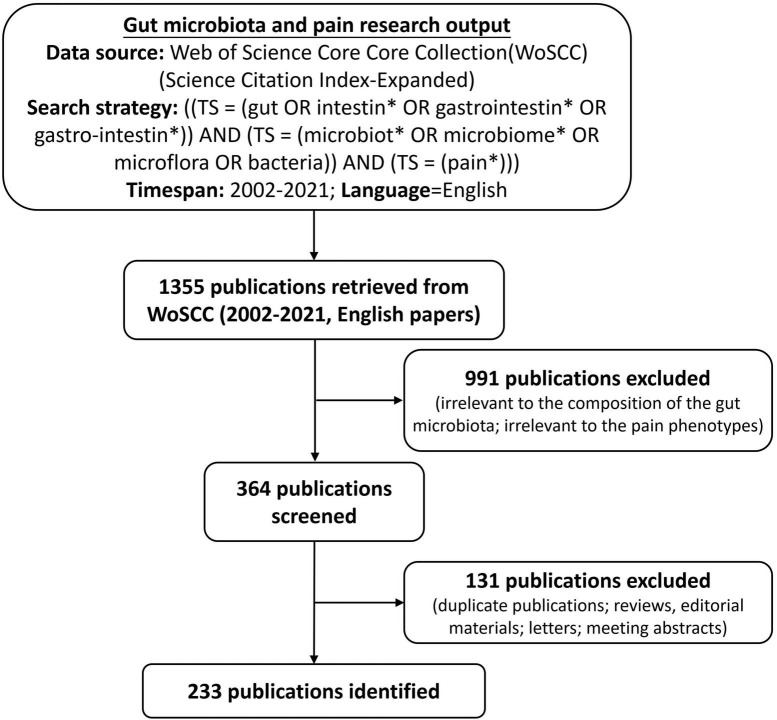
Flowchart for including and excluding publications.

Web of Science Core Collection (WoSCC) database is one of the best-known and high-quality databases (31). We conducted a comprehensive literature search within the Science Citation Index-Expanded (SCI-EXPANDED) of WoSCC database, using the following search strategy: [TS = (gut OR intestin* OR gastrointestin* OR gastrointestin*)] AND [TS = (microbiot* OR microbiome* OR microflora OR bacteria) AND (TS = (pain*))] from January 1, 2002, to December 31, 2021. The publication language was restricted to English. To avoid database update bias, we performed all data extraction and data downloads on the same day (April 22, 2022). To confirm the accuracy of bibliometric analysis results, we identified all publications retrieved by the search strategy above, including titles, abstracts, and publication years. The exclusion criteria were as follows: (i) irrelevant to the composition of the gut microbiota, (ii) irrelevant to the pain phenotypes, (iii) only articles were included, whereas other document types (e.g., reviews, editorial materials, letters, and meeting abstracts) were excluded, and (iv) duplicate publications were excluded. In total, 233 articles were ultimately analyzed in our study. The detailed screening is shown in Figure 1.

### Bibliometric analysis

In order to describe all literature characteristics about research on the links between the gut microbiota and pain, we converted all data that meet the requirements from WoSCC to txt format and imported them into the Online Analysis Platform of Literature Metrology^1^; Bibliometrix (32); CiteSpace V5.8 R3 (33) (Drexel University, Philadelphia, PA, USA), and VOSviewer 1.6.15 (34) (Leiden University, Leiden, The Netherlands) for further analysis.

Web of Science was used to retrieve target data and to analyze the publication trend by year and the data was imported into the R package ggplot2 to form a histogram. The website of bibliometrics, the Online Analysis Platform of Literature Metrology was used to show the annual publication trend from different countries and regions, the inter-country/region cooperative relationships, and the top 10 most cited journals. Moreover, the collaborations between institutions and those between authors were analyzed by the VOSviewer software.

Full records and cited references of the retrieved articles were downloaded from the WoSCC database and saved as.txt format for further analysis by the CiteSpace software (version 5.8 R3c). We set the following format: time slicing from January 2002 to December 2021, years per slice choosing 1. The selection used a modified g-index in each slice: *g2* ≤ *k*Σ_*i*_ ≤ *_*g*_c_*i*_, k ∈ Z^+^, k* = *25*.

As the most popular and recognized bibliometric visualization tool, CiteSpace was used to visualize the map of cooperation between countries/regions and between institutes, co-authorship, reference co-citation and to figure out the bursts of keywords between 2002 and 2021. The R package bibliometrix was used to output the top 100 high-frequency keywords as a word cloud. VOSviewer was used to visualize the inter-institution cooperation and co-authorship.

## Results

### Quantity and trends analysis of published papers

**FIGURE 2:**
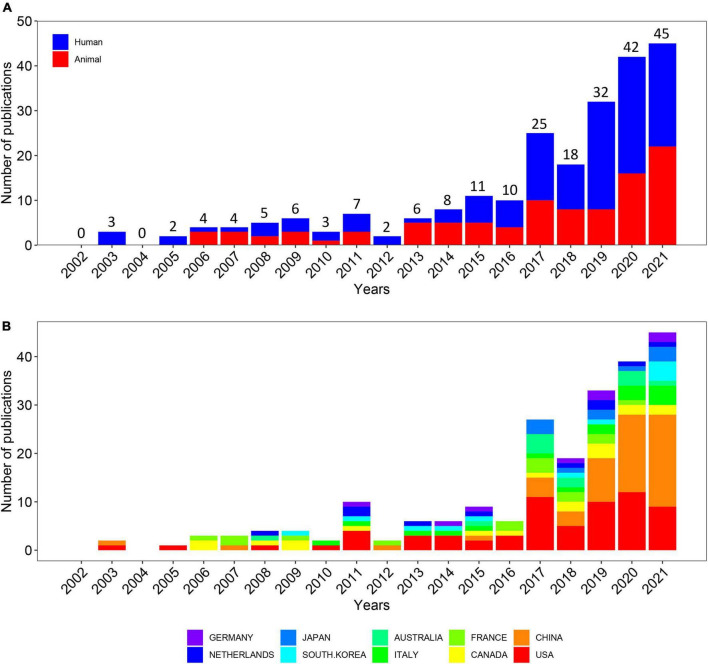
Quantity and trends analysis of published papers. **(A)** Number of annual research publications and growth trends on the topic of the links between the gut microbiota and pain from 2002 to 2021, export of results from web of sciences. The red color represents animal experimental studies and the blue color represents clinical trials. **(B)** Number of annual research publications and growth trends on the topic of the links between the gut microbiota and pain from 2002 to 2022, export of results from the Online Analysis Platform of Literature Metrology.

Out of 1,355 publications, the search retrieved 233 articles that met the inclusion and exclusion criteria (Figure 1). Over the past 20-year period, the development track showed two stages: one was the initial period (2002–2015), which had a very slow development speed, and the other was the rapid development period (2016–2021). The number of publication outputs increased from 3 publications in 2003 to 45 publications in 2021, and the compound annual growth rate (CAGR) was 16.24% (Figure 2A). Compound annual growth rate (CAGR) was the annualized average rate of growth between the years 2003 and 2021, calculated as follows: CAGR = [(value in the year 2021/value in the year 2003)^(1/18)^−1]. The number of publication outputs grew at a high CAGR of 35.10% from 2016 to 2021. Moreover, we used Microsoft Excel 2019 to build a growth trend model as follows: f(x) = ax^3^ + bx^2^ + cx + d, which predicted that nearly 100 articles will be published by 2025 (Supplementary Figure 1). Sixty-seven articles (28.76% of 233) were about IBS-related abdominal pain. The majority of the included articles (60.09% of 233) were about visceral pain (primary or secondary) in the abdominal region (Supplementary Table 1).

In order to figure out which countries or regions played leading roles on the links between the gut microbiota and pain during the past 20 years, the number of articles published by different countries and regions were counted on the website, the Online Analysis Platform of Bibliometrics.^2^ The histogram showed the number of publications from the top 10 countries/regions over the 20 years (Figure 2B). Overall, 233 articles were published by 51 countries and regions. Notably, the USA had long dominated the development of research on the links between the gut microbiota and pain while the number of publications from China first exceeded that of the USA in 2020 and maintained rapid growth in the last 4 years.

### Analysis of scientific collaboration network

**FIGURE 3:**
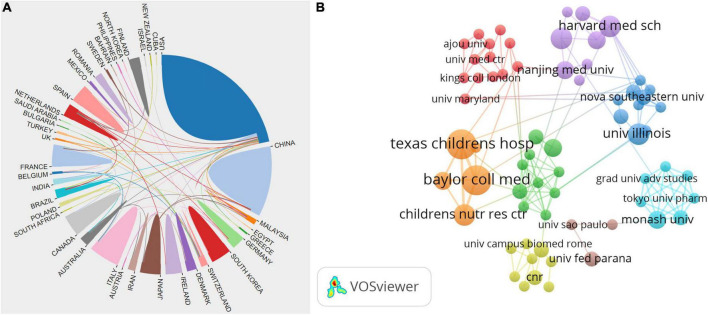
Visualization map of the scientific collaboration network analysis of gut microbiota research in the pain field from 2002 to 2021. Collaboration among countries/regions **(A)**, institutions **(B)**. The size of the concentric circle represented the number of articles published by each institution and the thickness of the connecting lines indicated the degree of cooperation between institutions. The brighter the color of the concentric circle is, the more productive the institution has been on the topic of the links between the gut microbiota and pain in recent years.

Collaboration network analysis can provide quantitative information for evaluating collaboration between countries, institutes, and scholars and identifying the key cooperators. Overall, 233 articles were published by 38 countries and regions from 2002 to 2021. We analyzed cooperative relationships between these countries using the bibliometrics online analysis platform (Figure 3A). The result shows that the USA was the country most frequently involved in international cooperation.

In order to clarify the inter-institutional cooperation in this field, we imported TXT files into the CiteSpace software. A total number of 558 institutes made contributions to microbiota research in the pain field. As shown in Figure 3B, the top 15 productive institutions were listed in the visualized graph, in which each concentric circle represented an institution and the links indicated the strength of institutional cooperation with each other. The size of the concentric circle represented the number of articles published by each institution and the thickness of the connecting lines indicated the degree of cooperation between institutions. The brighter the color of the concentric circle is, the more productive the institution has been on the topic of the links between the gut microbiota and pain in recent years. Amongst them, Baylor College of Medicine, McMaster University, Texas Children’s Hospital, and Institut National de la Recherche Agronomique (INRA) were the institutions that were the most frequently involved in international cooperation. Baylor College of Medicine, represented by the largest node marked with an orange ring dot, had the most publications and the most extensive cooperation, with more than 24 institutions, such as Children’s Nutrition Research Center, Texas Children’s Hospital, and Columbia University.

**FIGURE 4:**
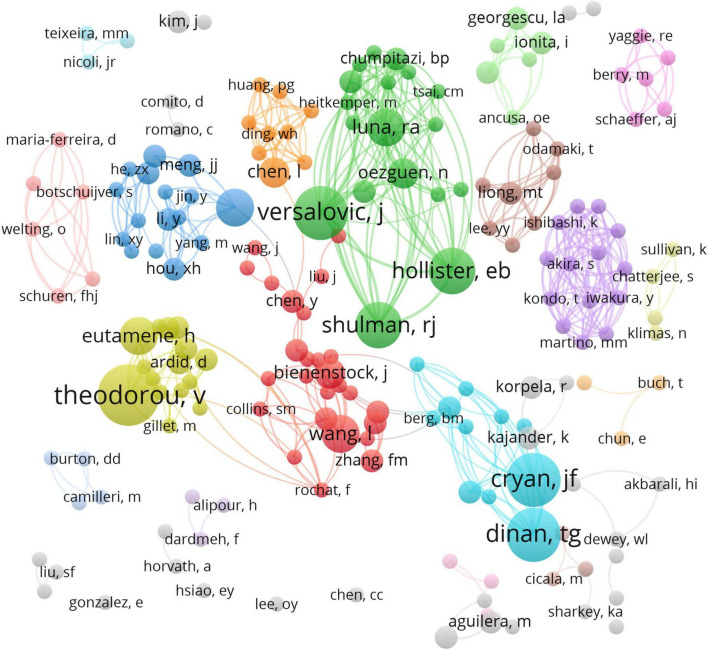
VOSviewer network of authorship in research on the links between the gut microbiota and pain. Each circle represents an author and the link between the two circles means a collaboration between each other.

A total of 1,777 authors were obtained in 233 articles. The co-authorship network map is shown in Figure 4, containing 188 nodes and 541 collaboration lines. Because the co-authorship network map only shows prolific authors (publications ≥ 2), it contains 188 nodes rather than 1,777 nodes. The nodes represent the authors and the link lines between them represent their cooperative relationships. However, all of the nodes have very low centrality (< 0.01). Represented by the largest node, Vassilia Theodorou is the Director of Research of the Neuro-Gastroenterology and Nutrition Unit at INRAE Toxalim, France. Prof. Theodorou’s research team has studied how *Lactobacillus farciminis* treatment suppresses stress-induced visceral hypersensitivity and *Bifidobacterium longum* and *Lactobacillus helveticus* synergistical therapeutic effect on stress-related visceral hypersensitivity. James Versalovic is the second most prolific author, who has 7 articles published in the past 20 years with 1,025 citations. James Versalovic, Robert J Shulman, and Emily B Hollister collaborate often and they all work at Baylor College of Medicine, one of the institutions which were the most frequently involved in international cooperation. Prof. Versalovic’s research team has studied how the gut microbiome impacts children’s health with the help of metagenomic medicine.

### Analysis of journals

Bibliometrics online analysis platform was used to analyze journal influence. The top 10 most-cited journals for the gut microbiota research in the pain field are presented in Table 1, which indicates that articles published in *Alimentary pharmacology and therapeutics* and *Gut* were cited most frequently with 57 times, during the past 20 years, followed by those in *Neurogastroenterology and Motility* (35), *Nature Medicine* (30), *Scientific Reports* (24), *Nature Neuroscience* (23), *Neuroscience* (21), *Proceedings of the National Academy of Sciences of the United States of America* (18), *Gastroenterology* (14), and *eLife* (11). All of the 10 journals were from the USA and the UK and had an IF above 3.0. All of these journals, except for *Neurogastroenterology and Motility* and *Neuroscience*, were in the first quartile (Q1) of the Journal Citation Report (JCR), indicating that these journals were influential ones and the research field gained its high recognition. The most productive journal was *Neurogastroenterology and Motility*, which published 11 articles. The paper published in *Nature Medicine* had the highest average citation per paper (30 times).

**TABLE 1:** The top 10 most-cited journals for gut microbiota research in the pain field.

Rank	Journal title	Paper numbers	Total citations	Average citation per paper	IF (2020)	Country	JCR
1	*Alimentary Pharmacology and Therapeutics*	8	57	7.13	8.171	UK	Q1
2	*Gut*	3	57	19.00	23.059	UK	Q1
3	*Neurogastroenterology and Motility*	11	42	3.82	3.598	UK	Q2
4	*Nature Medicine*	1	30	30.00	53.440	USA	Q1
5	*Scientific Reports*	8	24	3.00	4.379	UK	Q1
6	*Nature Neuroscience*	2	23	11.50	24.884	USA	Q1
7	*Neuroscience*	2	21	10.50	3.590	USA	Q3
8	*Proceedings of the National Academy of Sciences of the United States of America*	3	18	6.00	11.205	USA	Q1
9	*Gastroenterology*	3	14	4.67	22.681	USA	Q1
10	*eLife*	1	11	11.00	8.140	UK	Q1

### Analysis of number of citations

The number of citations is an important indicator of the impact of an article in a research area. The number of citations of these 233 articles was counted and ranked, and the top 10 are shown in Table 2. The most cited article was published in Microbiome in 2017, with 560 citations. This 7–8 weeks small open-label clinical trial evaluated the impact of Microbiota Transfer Therapy (MTT) on gut microbiota composition and GI and ASD symptoms of 18 ASD-diagnosed children. GI symptoms and behavioral ASD symptoms improved significantly and remained improved 8 weeks after the treatment ended (36). The second one was published in Nature Medicine in 2007, with 511 citations. Rousseaux et al. (26) found that specific Lactobacillus strains induced the expression of μ-opioid and cannabinoid receptors in intestinal epithelial cells, mediating analgesic functions similar to the effects of morphine. The third one demonstrated that bacteria can directly activate nociceptors for Staphylococcus aureus-induced pain in 2013 (37).

**TABLE 2:** The top 10 cited articles in the included 233 articles about the relationship between the gut microbiota and pain.

Rank	Title	Journal	Year	Cited frequency	Average citation per paper
1	Microbiota Transfer Therapy alters gut ecosystem and improves gastrointestinal and autism symptoms: an open-label study	*Microbiome*	2017	560	93.33
2	Lactobacillus acidophilus modulates intestinal pain and induces opioid and cannabinoid receptors	*Nature Medicine*	2007	511	31.94
3	Bacteria activate sensory neurons that modulate pain and inflammation	*Nature*	2013	465	46.5
4	Gastrointestinal microbiome signatures of pediatric patients with irritable bowel syndrome	*Gastroenterology*	2011	396	33
5	A randomized controlled trial of a probiotic, VSL#3, on gut transit and symptoms in diarrhea-predominant irritable bowel syndrome	*Alimentary Pharmacology and Therapeutics*	2003	320	16
6	Specific probiotic therapy attenuates antibiotic induced visceral hypersensitivity in mice	*Gut*	2006	300	17.65
7	A randomized controlled trial of a probiotic combination VSL# 3 and placebo in irritable bowel syndrome with bloating	*Neurogastroenterology and Motility*	2005	265	14.72
8	TRPA1 channels mediate acute neurogenic inflammation and pain produced by bacterial endotoxins	*Nature Communications*	2014	259	28.78
9	A probiotic mixture alleviates symptoms in irritable bowel syndrome patients: a controlled 6-month intervention	*Alimentary Pharmacology and Therapeutics*	2005	239	13.28
10	Clinical trial: multispecies probiotic supplementation alleviates the symptoms of irritable bowel syndrome and stabilizes intestinal microbiota	*Alimentary Pharmacology and Therapeutics*	2008	227	15.13

### Analysis of co-citation references and clustered network

**FIGURE 5:**
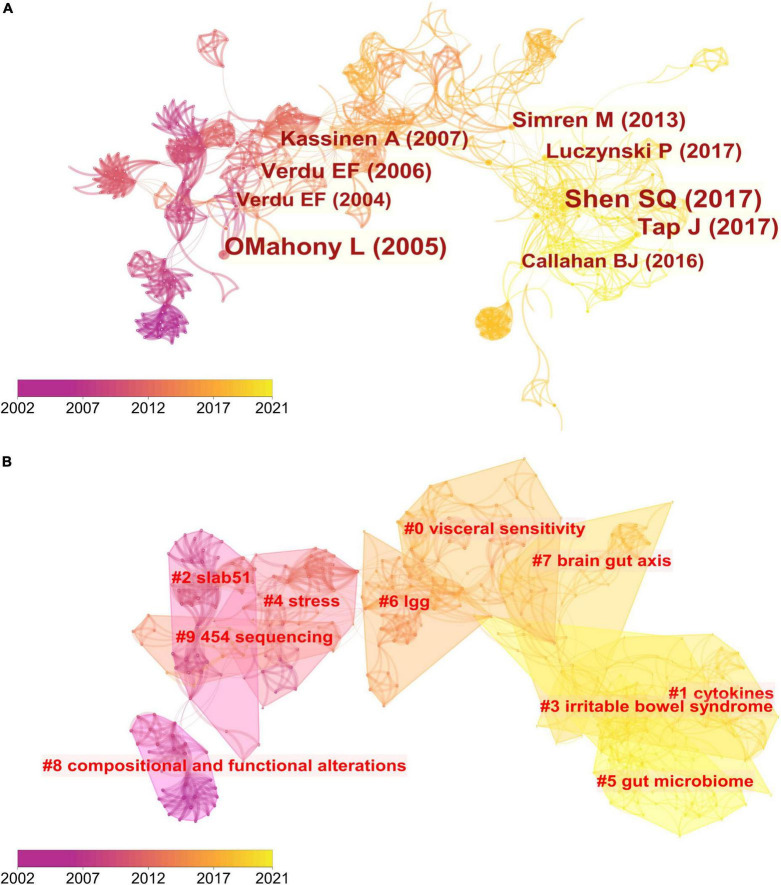
Reference co-citation network analysis of publications on the links between the gut microbiota and pain between 2002 and 2021. **(A)** Cluster visualization of the reference co-citation map. The top 10 largest clusters of citing articles are shown. **(B)** Cluster visualization according to keywords generated from the references by CiteSpace.

Co-cited references are those co-cited in the reference lists of other articles. We retrieved a total of 233 articles and their 570 references from the Science Citation Index-Expanded (SCI-EXPANDED) of the Web of Science Core Collection (WoSCC) database and analyzed them by the CiteSpace to cluster them. The map of co-citation reference in the CiteSpace on the links between the gut microbiota and pain were presented in Figure 5A. Each node represents a reference and the size of the node is positively related to the frequency of citation. The link between nodes means these articles were cited as references in the same article and line thickness means the correlation with the co-cited papers. The redder nodes (left) represent references cited in earlier years while the yellower ones (right) represent papers that have been frequently cited in recent years. Because many references shared the tenth place with each other, Table 3 only presented the top 9 co-cited references related to the gut microbiota research in the pain field.

**TABLE 3:** The top 9 co-cited references related to the gut microbiota research in the pain field between 2002 and 2021.

Rank	Title	First author	Journal	Year	Cited frequency	DOI	Cluster ID
1	Gut microbiota is critical for the induction of chemotherapy-induced pain.	Shen SQ	*Nat Neurosci*	2017	15	https://doi.org/10.1038/nn.4606	3
2	*Lactobacillus* and *Bifidobacterium* in irritable bowel syndrome: symptom responses and relationship to cytokine profiles	OMahony L	*Gastroenterology*	2005	13	https://doi.org/10.1053/j.gastro.2004.11.050	4
3	Identification of an Intestinal Microbiota Signature Associated With Severity of Irritable Bowel Syndrome	Tap J	*Gastroenterology*	2017	11	https://doi.org/10.1053/j.gastro.2016.09.049	5
4	Intestinal microbiota in functional bowel disorders: a Rome foundation report	Simren M	*Gut*	2013	9	https://doi.org/10.1136/gutjnl-2012-302167	0
5	Specific probiotic therapy attenuates antibiotic induced visceral hypersensitivity in mice	Verdu EF	*Gut*	2006	9	https://doi.org/10.1136/gut.2005.066100	4
6	Microbiota regulates visceral pain in the mouse	Luczynski P	*eLife*	2017	8	https://doi.org/10.7554/eLife.25887	3
7	The fecal microbiota of irritable bowel syndrome patients differs significantly from that of healthy subjects	Kassinen A	*Gastroenterology*	2007	8	https://doi.org/10.1053/j.gastro.2007.04.005	4
8	DADA2: High-resolution sample inference from Illumina amplicon data	Callahan BJ	*NAT Methods*	2016	7	https://doi.org/10.1038/nmeth.3869	3
9	Lactobacillus paracasei normalizes muscle hypercontractility in a murine model of post-infective gut dysfunction	Verdu EF	*Gastroenterology*	2004	7	https://doi.org/10.1053/j.gastro.2004.06.007	4

The map of co-citation clustered according to keywords generated from the references by CiteSpace is shown in Figure 5B. The timeline visualization of the reference co-citation map contained 570 nodes and 2,009 links, representing the cited references and their co-cited relationships, respectively. The number of cluster tags is reversely correlated with the number of articles that each cluster included. The analysis of co-citation clusters revealed the most relevant terms on the links between the gut microbiota and pain, which included #0 visceral sensitivity, #1 cytokines, #2 slab51, #3 irritable bowel syndrome, #4 stress, #5 gut microbiome, #6 lgg, #7 brain-gut axis, #8 SLAB51, and #9 454 sequencing. *Lactobacillus rhamnosus* GG (LGG) increases treatment success in children with abdominal pain-related functional gastrointestinal disorders (38). SLAB51 is a probiotic formulation that markedly reduced oxidative stress in AD mice brains (39). As shown in Table 3, 4 of the top 9 co-cited references were in cluster #4 stress and 3 of the top 9 ones were in cluster #3 irritable bowel syndrome.

### Analysis of co-occurring keywords and burst detection

**FIGURE 6:**
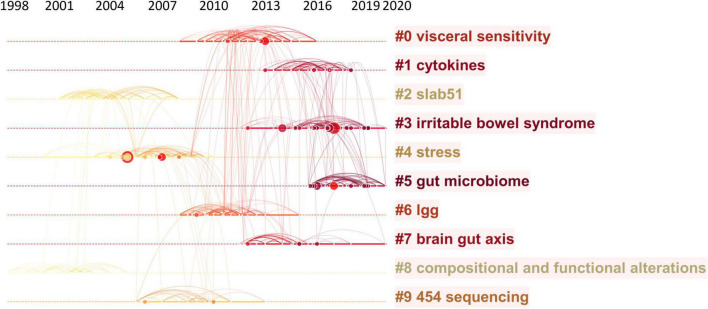
Timeline visualization of the reference co-citation map. Large nodes or nodes with red tree-rings are either highly cited or have citation bursts in a given time slice. Right side = cluster labels.

To map the knowledge structure of research, Figure 6 shows a timeline view of the keyword co-occurrence network. Each circle represents a main cited paper in a certain cluster and the citation tree-rings of different sizes on the timeline represent citation rates. Large nodes or nodes with red tree-rings are either highly cited or have citation bursts in a given time slice. Visceral sensitivity, cytokines, slab51, irritable bowel syndrome, stress, gut microbiome, lgg, brain-gut axis, compositional and functional alternations, and 454 sequencing were the top 10 clusters in co-occurrence cluster analysis. Cluster #3 irritable bowel syndrome and cluster #4 stress had the highest degree of citation bursts.

**FIGURE 7:**
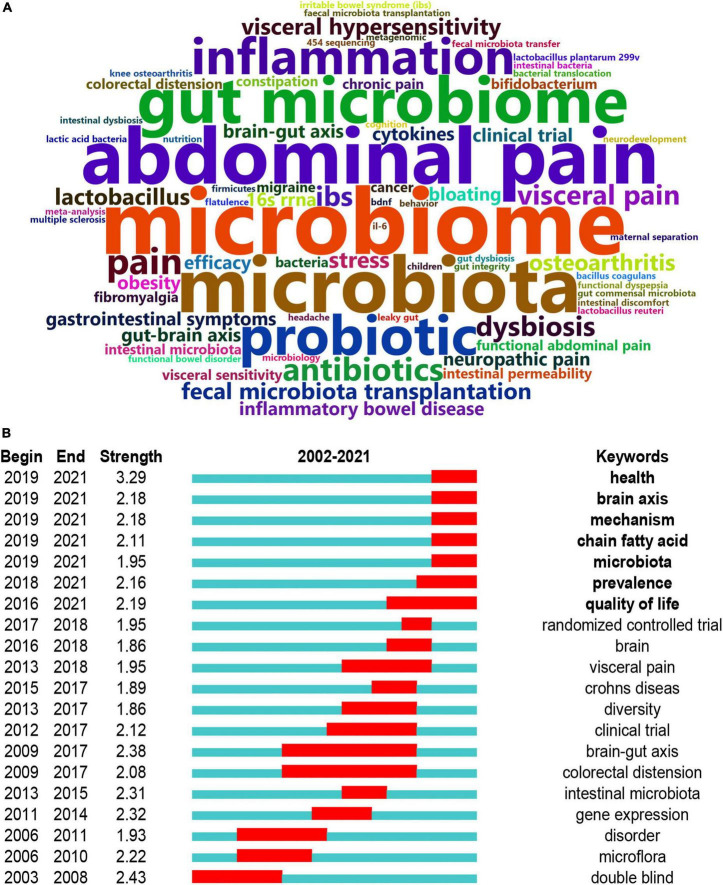
Analysis of keywords. **(A)** The word cloud analysis of the top 50 high-frequency keywords on the links between the gut microbiota and pain between 2002 and 2021. **(B)** Keywords with the strongest citation bursts in original articles on the links between the gut microbiota and pain. Keyword marked in red indicates a sudden increase in usage frequency of this keyword during that period. Blue represents a relatively unpopular time period.

The data were further imported into “Bibliometrix” (R package) to output the top 100 high-frequency keywords as a word cloud. As illustrated in Figure 7A, “microbiome,” “microbiota,” “abdominal pain,” “gut microbiome,” “probiotic,” and “inflammation” had the highest frequency. Burst detection can be used to predict new frontier topics in research in a particular field. Figure 7B shows the top 20 keywords with the strongest citation bursts. Keyword marked in red indicates a sudden increase in usage frequency of this keyword during that period. Blue represents a relatively unpopular time period. “Health” was the strongest burst keyword (strength 3.29) in this field from 2002 to 2021 and was followed by “double blind” (strength 2.43), “brain-gut axis” (strength 2.38), and “gene expression” (strength 2.32). “Health,” “brain axis,” “mechanism,” “chain fatty acid,” “microbiota,” “prevalence,” and “quality of life” were the current research hot spots. By querying the abstract of the included articles, “chain fatty acid” was identified as short-chain fatty acids (SCFAs).

## Discussion

This is the first bibliometric and visual analysis of research on the links between the gut microbiota and pain from 2002 to 2021. Compared with traditional reviews, an analysis based on bibliometric tools provides a better insight into the evolving research foci and trends, and this type of data analysis is comparatively more comprehensive and objective. This study included 233 articles from the Science Citation Index-Expanded (SCI-EXPANDED) of the Web of Science Core Collection (WoSCC) database, and each publication retrieved was completely relevant to the topic. The number of publication outputs increased rapidly since 2016. The collaboration network revealed that the USA, Baylor College of Medicine, Vassilia Theodorou were the most influential country, institute, and scholar, respectively. Keyword burst detection indicated that the brain-gut axis and chain fatty acid were the current research hotspots. Short-chain fatty acids (SCFAs), as metabolites of the gut microbiota, play an important part in the brain-gut axis. Short-chain fatty acids-mediated mechanism of pain regulation will be a research direction of great importance on the links between the gut microbiota and pain. Our timely analysis of the hotpots and research trends may promote the development of this field.

There was rapid growth in the number of articles on the links between the gut microbiota and pain since 2016, growing at a CAGR of 35.10%. In Falony et al. and Zhernakova et al. targeted general populations in Belgium and the Netherlands, respectively, and completed the first metagenomics analysis of the gut microbiome of over 1,000 participants (40, 41). Subsequently, three studies found attributes of the genetic architecture for microbiome traits by genome-wide association analysis (42), marking a new phase of microbial whole-genome resequencing. According to the number of published papers, the USA had long dominated the development of the chosen field. However, China has exceeded the USA since 2020.

Collaboration network analysis can provide quantitative information for evaluating collaboration between countries, institutes, and scholars and identifying the key cooperators. The analysis of scientific collaboration network showed that the USA, Baylor College of Medicine, and Vassilia Theodorou were the most influential country, institute, and scholar, respectively. The USA had a huge advantage in this field, possibly owing to the long-standing leading role the USA played in bioscience. The other reason was the crucial financial support from research funds. For example, the US National Institute of Mental Health (NIMH) funded seven pilot studies with up to US$1 million each from 2013 to 2015 and the US Office of Naval Research pumped around US$14.5 million into work examining the gut microbiota-brain axis (35).

Analysis of journals can help researchers select appropriate journals for submission. Among the top 10 most cited journals, *Alimentary pharmacology and therapeutics* and *Gut* possessed the most citations (43). *Gut* earned its reputation for publishing first-class clinical research in the relevant research field. Moreover, articles published on *Gut* had more average citations per paper (19.00 times) than articles published on *Alimentary pharmacology and therapeutics* (7.13 times). Through our research, we found that the top 10 most cited journals published less than a quarter (18.03%) of the total articles on the links between the gut microbiota and pain. The paper published in *Nature Medicine* had the highest average citation per paper, which discovered that oral administration of *Lactobacillus acidophilus* induced the expression of μ-opioid and cannabinoid receptors and mediated analgesic effects (26).

Among the top 10 articles with the highest number of citations, preclinical research accounted for 4/10 of the total. Two articles found that the administration of *Lactobacillus* normalized visceral sensitivity (25, 26), and more specifically, Rousseaux et al. found that *Lactobacillus acidophilus*-mediated analgesic functions by inducing the expression of μ-opioid and cannabinoid receptors in intestinal epithelial cells (26). Meseguer et al. proved that TRPA1 is a molecular determinant of direct LPS effects on nociceptors (44). In the six clinical trials, four articles (45–48) investigated the effects of probiotics (e.g., VSL# 3) on patients with IBS and one article found microbiota transfer therapy improved gastrointestinal and autism symptoms in patients with Autism Spectrum Disorders (ASD) (36). Overall, these articles focused on visceral pain and the peripheral mechanism underlying the regulation of pain by the gut microbiota.

According to co-citation references analysis, 4 in the top 9 co-cited references were published on *Gastroenterology* and the majority (5/9) of these articles expounded on the role of intestinal microbiota played in functional bowel disorders (49–53). The most co-cited reference was a brief communication published on *Nature Neuroscience* in 2017 (28). Researchers found that oxaliplatin-induced mechanical hyperalgesia was reduced in germ-free mice and in mice pretreated with antibiotics, and these effects appeared to be mediated, in part, by TLR4 expressed on hematopoietic cells. The second most-cited paper was published on *Gastroenterology* in 2005 (52). It was a clinical trial evaluating the response of probiotic preparations containing a *Lactobacillus* or *Bifidobacterium* in patients with IBS. Abdominal pain was reduced in the two treatments and the symptomatic changes were associated with a normalized ratio of anti-inflammatory to pro-inflammatory cytokine. The third-ranked publication was published on *Gastroenterology* in 2017. Although the study found no differences in fecal microbiota between IBS vs. healthy individuals regarding α-diversity or β-diversity at any taxonomy level, differences between patients with IBS and healthy subjects were observed based on enterotype distribution, with IBS being more likely to belong to the *Bacteroides* enterotype. IBS symptom severity was associated with microbial richness, presence of methanogens, and enterotypes enriched with *Clostridiales* or *Prevotella* species (49).

Co-citation cluster visualization showed that four articles of the top co-citation were located in the second largest theme cluster #1, “fibromyalgia.” These articles were published in 2016 or 2017, indicating that fibromyalgia possibly emerged as a new disease model to probe the links between the gut microbiota and pain in recent 5 years. Fibromyalgia is a complex disease characterized by chronic widespread musculoskeletal pain (54). Minerbi et al. observed an alteration in butyrate-metabolizing species in individuals with fibromyalgia and demonstrated gut microbiome alteration in non-visceral pain for the first time (55). The dynamics of the field were partly characterized by references with citation bursts. Cluster #3 irritable bowel syndrome and cluster #4 stress had the highest degree of citation bursts.

The word cloud produced by the R package “Bibliometrix” illustrated the high-frequency keywords. Accordingly, research on the links between the gut microbiota and pain mainly focused on abdominal pain, a kind of visceral pain. Supplementary Table 1 is also supported this view. In the International Classification of Diseases, 11th Revision (ICD-11), there were many types of chronic pain except for chronic primary/secondary visceral pain, such as chronic cancer related pain, chronic postsurgical or post-traumatic pain, chronic secondary musculoskeletal pain, and chronic neuropathic pain. Therefore, the links between the gut microbiota and pain should receive more attention and separate consideration based on the types of pain.

According to the co-occurring keyword analysis, we identified some hotspots of great importance, including brain-gut axis, short-chain fatty acid, visceral pain, and double-blind randomized controlled trial. Half of the 20 keywords began between 2015 and 2019, which was probably related to the publication outputs burst since 2016. “Health,” “brain axis,” “mechanism,” “chain fatty acid,” “microbiota,” “prevalence,” and “quality of life” were the current research hotspots. “Health,” “mechanism,” “prevalence,” and “quality of life” are of little practical significance and “brain axis” means brain-gut axis. Based on the above information, the current research focused on the brain-gut axis and short-chain fatty acid. The bidirectional communication between the gut and the brain, namely the gut-brain axis, involves multiple pathways including immune, neural, endocrine, and metabolic routes. Short-chain fatty acids (SCFAs) are derived primarily from bacterial fermentation of carbohydrates and proteins. These two words showed that previous studies are based on the research paradigm of the gut-brain axis and focused on metabolic routes, rather than other pathways in the gut-brain axis. In the absence of the gut microbiota, there was decreased production of short-chain fatty acids that were necessary for adequate inflammasome assembly and IL-1β production (56). Short-chain fatty acids (SCFAs) regulate leucocyte functions, such as the production of cytokines (TNF-α, IL-2, IL-6, and IL-10), eicosanoids, and chemokines (CCL2) (23). For example, butyrate enhances the release of the anti-inflammatory cytokine IL-10 and sodium butyrate treatment attenuates pain attacks in a mouse model of nitroglycerine (NTG)-induced migraine (43, 57). Interestingly, there are conflicting reports about the function of SCFAs. Bourdu et al. (58) showed that butyrate might induce colonic hypersensitivity in rats without altered pathology, while Vanhoutvin et al. (59) showed a significant decrease in visceral perception and pain in healthy subjects. It is possible that the dose of butyrate administered determines its effect, butyrate at physiologically relevant concentrations might decrease colonic hypersensitivity, whereas a higher concentration might produce an opposite effect (60).

In addition to SCFAs, other types of microbial metabolites also remarkably affect pain signaling. *Lactobacillus* spp., *Bifidobacterium dentium*, and *Bifidobacterium* spp. can produce GABA, a chief inhibitory neurotransmitter (61). The activation of the GABA_*A*_ receptor on DRG neurons by GABA depolarized the majority of sensory neuronal soma and produced a net inhibitory effect on the nociceptive transmission (62). 5-HT, another important neurotransmitter, can be produced by *Candida* spp., *Streptococcus* spp., *Escherichia* spp., and *Enterococcus* spp. The effects of 5-HT on primary nociceptive neurons depend on the subfamilies of activated 5-HT receptors, producing either hyperpolarizing or depolarizing effects (63). Polyunsaturated fatty acids (PUFAs), another intestinal microbial metabolite, are endogenous agonists of TRPV4 that lead to peripheral hypersensitivity (10).

Targeting the gut microbiota in chronic pain, there are experimental therapies under preclinical and clinical trials, such as probiotics, low-FODMAP intervention, and fecal microbiota transplantation (FMT). However, there are controversial results. Some claimed that antibiotics improve the condition of neuropathic pain while others illustrated antibiotics induce hyperalgesia (64). Besides, treatment options for visceral pain are often unsatisfactory, potentially due to the multiple factors that affect the perception and maintenance of this type of pain (65). Furthermore, it should be noted that much of what we know about the mechanisms of pain derived from somatic experimental studies, rather than visceral nociception (66). The neurological mechanisms of visceral pain vary from those of somatic pain, and the differences are relevant to clinical management.

This study is not without its limitations. First, because the current version of CiteSpace only supports data from the WoSCC database, all the publications were extracted from the WoSCC database rather than other databases, such as PubMed or Embase, which may have bias. Besides, our search strategy may not have included all the relevant articles because of the limited search terms, types of literature, and languages. However, considering the dominant position of the English language in the international academic communication and the comprehensiveness of the search terms, we believe that our search strategy included most of the relevant articles. Therefore, we consider that our findings offer a bird’s-eye view of the overall research and reflect the general trend in this field. Furthermore, although the number of articles published in the field has surged in recent years, the overall number is still relatively small. Hence, this study was not so powered to detect hotspots and research trends, owing to the relatively small size of included publications. Since the number of publications has seen rapid growth and nearly 100 articles will be published by 2025 according to our prediction, it is meaningful to present a bibliometric review and predict research hotspots in this emerging field. It should be noted that citation number may favor older articles and there are many factors that affect citation rates, such as self-citations and incomplete citing (67). These limitations should be noted and avoided as much as possible in future studies.

## Conclusion

This is the first bibliometric and visual analysis of research on the links between the gut microbiota and pain. With the help of bibliometric mapping, we analyzed research on the links between the gut microbiota and pain over the past 20 years. The number of publications has seen rapid growth since 2016. The USA was the most active country in international scientific cooperation. The current research focused on the brain-gut axis and chain fatty acid. Accordingly, the mechanism of gut microbial metabolites regulate pain through the brain-gut axis will be a research direction of great importance. This study provides instructive assistance to direct future research efforts, which may be helpful to discover the mechanism and new treatment of pain.

## Data availability statement

The original contributions presented in this study are included in the article/Supplementary material, further inquiries can be directed to the corresponding authors.

## Author contributions

LY and PG raised the conception of the study and designed the study. MM screened articles and wrote the original manuscript. YJ confirmed the accuracy of the search results. YZ conducted the CiteSpace and VOSviewer analysis. LY, PG, SY, WY, CC, and YJ revised the manuscript and edited critically. All authors contributed to the article and approved the submitted version.

## Footnotes

1
https://bibliometric.com/app

2
http://bibliometric.com/

## References

[1] Raja SN Carr DB Cohen M Finnerup NB Flor H Gibson S The revised international association for the study of pain definition of pain: concepts, challenges, and compromises. *Pain.* (2020) 161:1976–82. 10.1097/j.pain.0000000000001939 32694387 PMC7680716

[2] Zimmer Z Fraser K Grol-Prokopczyk H Zajacova AA. Global study of pain prevalence across 52 countries: examining the role of country-level contextual factors. *Pain.* (2022) 163:1740–50. 10.1097/j.pain.0000000000002557 35027516 PMC9198107

[3] O’Connell NE Ferraro MC Gibson W Rice AS Vase L Coyle D Implanted spinal neuromodulation interventions for chronic pain in adults. *Cochrane Database Syst Rev.* (2021) 12:CD013756. 10.1002/14651858.CD013756.pub2 34854473 PMC8638262

[4] Cohen SP Vase L Hooten WM. Chronic pain: an update on burden, best practices, and new advances. *Lancet.* (2021) 397:2082–97. 10.1016/S0140-6736(21)00393-7 34062143

[5] Wu A Dong W Liu S Cheung JPY Kwan KYH Zeng X The prevalence and years lived with disability caused by low back pain in China, 1990 to 2016: findings from the global burden of disease study 2016. *Pain.* (2019) 160:237–45. 10.1097/j.pain.0000000000001396 30234697 PMC6319591

[6] Haumann J Joosten EBA Everdingen M. Pain prevalence in cancer patients: status quo or opportunities for improvement? *Curr Opin Support Palliat Care.* (2017) 11:99–104. 10.1097/SPC.0000000000000261 28306569

[7] Bergman S Herrstrom P Hogstrom K Petersson IF Svensson B Jacobsson LT. Chronic musculoskeletal pain, prevalence rates, and sociodemographic associations in a Swedish population study. *J Rheumatol.* (2001) 28:1369–77. 11409133

[8] Volkow ND McLellan AT. Opioid abuse in chronic pain–misconceptions and mitigation strategies. *N Engl J Med.* (2016) 374:1253–63. 10.1056/NEJMra1507771 27028915

[9] Pusceddu MM Gareau MG. Visceral pain: gut microbiota, a new hope? *J Biomed Sci.* (2018) 25:73. 10.1186/s12929-018-0476-7 30309367 PMC6182804

[10] Lin B Wang Y Zhang P Yuan Y Zhang Y Chen G. Gut microbiota regulates neuropathic pain: potential mechanisms and therapeutic strategy. *J Headache Pain.* (2020) 21:103. 10.1186/s10194-020-01170-x 32807072 PMC7433133

[11] Agirman G Hsiao EY. Snapshot: the microbiota-gut-brain axis. *Cell.* (2021) 184:2524–e1. 10.1016/j.cell.2021.03.022 33930299

[12] Mayneris-Perxachs J Castells-Nobau A Arnoriaga-Rodriguez M Martin M de la Vega-Correa L Zapata C Microbiota alterations in proline metabolism impact depression. *Cell Metab.* (2022) 34:681–701e10. 10.1016/j.cmet.2022.04.001 35508109

[13] Du Toit A. Gut microbiota and depression. *Nat Rev Microbiol.* (2022) 20:190. 10.1038/s41579-022-00703-2 35169287

[14] Nikolova VL Hall MRB Hall LJ Cleare AJ Stone JM Young AH. Perturbations in gut microbiota composition in psychiatric disorders: a review and meta-analysis. *JAMA Psychiatry.* (2021) 78:1343–54. 10.1001/jamapsychiatry.2021.2573 34524405 PMC8444066

[15] Wang X Sun G Feng T Zhang J Huang X Wang T Sodium oligomannate therapeutically remodels gut microbiota and suppresses gut bacterial amino acids-shaped neuroinflammation to inhibit Alzheimer’s disease progression. *Cell Res.* (2019) 29:787–803. 10.1038/s41422-019-0216-x 31488882 PMC6796854

[16] Kim MS Kim Y Choi H Kim W Park S Lee D Transfer of a healthy microbiota reduces amyloid and tau pathology in an alzheimer’s disease animal model. *Gut.* (2020) 69:283–94. 10.1136/gutjnl-2018-317431 31471351

[17] Lou M Cao A Jin C Mi K Xiong X Zeng Z Deviated and early unsustainable stunted development of gut microbiota in children with autism spectrum disorder. *Gut.* (2021) 71:1588–99. 10.1136/gutjnl-2021-325115 34930815 PMC9279844

[18] Sharon G Cruz NJ Kang DW Gandal MJ Wang B Kim YM Human Gut Microbiota from Autism Spectrum Disorder Promote Behavioral Symptoms in Mice. *Cell.* (2019) 177:1600–18e17. 10.1016/j.cell.2019.05.004 31150625 PMC6993574

[19] Sampson TR Debelius JW Thron T Janssen S Shastri GG Ilhan ZE Gut microbiota regulate motor deficits and neuroinflammation in a model of Parkinson’s disease. *Cell.* (2016) 167:1469–80e12. 10.1016/j.cell.2016.11.018 27912057 PMC5718049

[20] Hou YF Shan C Zhuang SY Zhuang QQ Ghosh A Zhu KC Gut microbiota-derived propionate mediates the neuroprotective effect of osteocalcin in a mouse model of Parkinson’s disease. *Microbiome.* (2021) 9:34. 10.1186/s40168-020-00988-6 33517890 PMC7849090

[21] Ji RR Xu ZZ Gao YJ. Emerging targets in neuroinflammation-driven chronic pain. *Nat Rev Drug Discov.* (2014) 13:533–48. 10.1038/nrd4334 24948120 PMC4228377

[22] Ji RR Chamessian A Zhang YQ. Pain regulation by non-neuronal cells and inflammation. *Science.* (2016) 354:572–7. 10.1126/science.aaf8924 27811267 PMC5488328

[23] Guo R Chen LH Xing C Liu T. Pain regulation by gut microbiota: molecular mechanisms and therapeutic potential. *Br J Anaesth.* (2019) 123:637–54. 10.1016/j.bja.2019.07.026 31551115

[24] Aguilera M Cerda-Cuellar M Martinez V. Antibiotic-induced dysbiosis alters host-bacterial interactions and leads to colonic sensory and motor changes in mice. *Gut Microbes.* (2015) 6:10–23. 10.4161/19490976.2014.990790 25531553 PMC4615720

[25] Verdu EF Bercik P Verma-Gandhu M Huang XX Blennerhassett P Jackson W Specific probiotic therapy attenuates antibiotic induced visceral hypersensitivity in mice. *Gut.* (2006) 55:182–90. 10.1136/gut.2005.066100 16105890 PMC1856497

[26] Rousseaux C Thuru X Gelot A Barnich N Neut C Dubuquoy L Lactobacillus acidophilus modulates intestinal pain and induces opioid and cannabinoid receptors. *Nat Med.* (2007) 13:35–7. 10.1038/nm1521 17159985

[27] Savignac HM Kiely B Dinan TG Cryan JF. Bifidobacteria exert strain-specific effects on stress-related behavior and physiology in Balb/C mice. *Neurogastroenterol Motil.* (2014) 26:1615–27. 10.1111/nmo.12427 25251188

[28] Shen S Lim G You Z Ding W Huang P Ran C Gut microbiota is critical for the induction of chemotherapy-induced pain. *Nat Neurosci.* (2017) 20:1213–6. 10.1038/nn.4606 28714953 PMC5575957

[29] Amaral FA Sachs D Costa VV Fagundes CT Cisalpino D Cunha TM Commensal microbiota is fundamental for the development of inflammatory pain. *Proc Natl Acad Sci U.S.A.* (2008) 105:2193–7. 10.1073/pnas.0711891105 18268332 PMC2538897

[30] Chen C Hu Z Liu S Tseng H. Emerging trends in regenerative medicine: a scientometric analysis in citespace. *Expert Opin Biol Ther.* (2012) 12:593–608. 10.1517/14712598.2012.674507 22443895

[31] Guo Y Yang Y Xu M Shi G Zhou J Zhang J Trends and developments in the detection of pathogens in central nervous system infections: a bibliometric study. *Front Cell Infect Microbiol.* (2022) 12:856845. 10.3389/fcimb.2022.856845 35573778 PMC9100591

[32] Aria M Cuccurullo C. Bibliometrix : an R-tool for comprehensive science mapping analysis. *J Informetrics.* (2017) 11:959–75. 10.1016/j.joi.2017.08.007

[33] Du SH Zheng YL Zhang YH Wang MW Wang XQ. The last decade publications on diabetic peripheral neuropathic pain: a bibliometric analysis. *Front Mol Neurosci.* (2022) 15:854000. 10.3389/fnmol.2022.854000 35493329 PMC9043347

[34] van Eck NJ Waltman L. Software survey: vosviewer, a computer program for bibliometric mapping. *Scientometrics.* (2010) 84:523–38. 10.1007/s11192-009-0146-3 20585380 PMC2883932

[35] Smith PA. The tantalizing links between gut microbes and the brain. *Nature.* (2015) 526:312–4. 10.1038/526312a 26469024

[36] Kang DW Adams JB Gregory AC Borody T Chittick L Fasano A Microbiota transfer therapy alters gut ecosystem and improves gastrointestinal and autism symptoms: an open-label study. *Microbiome.* (2017) 5:10. 10.1186/s40168-016-0225-7 28122648 PMC5264285

[37] Chiu IM Heesters BA Ghasemlou N Von Hehn CA Zhao F Tran J Bacteria activate sensory neurons that modulate pain and inflammation. *Nature.* (2013) 501:52–7. 10.1038/nature12479 23965627 PMC3773968

[38] Horvath A Dziechciarz P Szajewska H. Meta-analysis: *Lactobacillus* rhamnosus Gg for abdominal pain-related functional gastrointestinal disorders in childhood. *Aliment Pharmacol Ther.* (2011) 33:1302–10. 10.1111/j.1365-2036.2011.04665.x 21507030

[39] Bonfili L Cecarini V Cuccioloni M Angeletti M Berardi S Scarpona S Slab51 probiotic formulation activates Sirt1 pathway promoting antioxidant and neuroprotective effects in an Ad mouse model. *Mol Neurobiol.* (2018) 55:7987–8000. 10.1007/s12035-018-0973-4 29492848 PMC6132798

[40] Falony G Joossens M Vieira-Silva S Wang J Darzi Y Faust K Population-level analysis of gut microbiome variation. *Science.* (2016) 352:560–4. 10.1126/science.aad3503 27126039

[41] Zhernakova A Kurilshikov A Bonder MJ Tigchelaar EF Schirmer M Vatanen T Population-based metagenomics analysis reveals markers for gut microbiome composition and diversity. *Science.* (2016) 352:565–9. 10.1126/science.aad3369 27126040 PMC5240844

[42] Benson AK. The gut microbiome-an emerging complex trait. *Nat Genet.* (2016) 48:1301–2. 10.1038/ng.3707 27787511

[43] Vinolo MA Rodrigues HG Nachbar RT Curi R. Regulation of inflammation by short chain fatty acids. *Nutrients.* (2011) 3:858–76. 10.3390/nu3100858 22254083 PMC3257741

[44] Meseguer V Alpizar YA Luis E Tajada S Denlinger B Fajardo O Trpa1 channels mediate acute neurogenic inflammation and pain produced by bacterial endotoxins. *Nat Commun.* (2014) 5:3125. 10.1038/ncomms4125 24445575 PMC3905718

[45] Kajander K Hatakka K Poussa T Farkkila M Korpela RA. Probiotic mixture alleviates symptoms in irritable bowel syndrome patients: a controlled 6-month intervention. *Aliment Pharmacol Ther.* (2005) 22:387–94. 10.1111/j.1365-2036.2005.02579.x 16128676

[46] Kajander K Myllyluoma E Rajilic-Stojanovic M Kyronpalo S Rasmussen M Jarvenpaa S Clinical trial: multispecies probiotic supplementation alleviates the symptoms of irritable bowel syndrome and stabilizes intestinal microbiota. *Aliment Pharmacol Ther.* (2008) 27:48–57. 10.1111/j.1365-2036.2007.03542.x 17919270

[47] Kim HJ Camilleri M McKinzie S Lempke MB Burton DD Thomforde GM A randomized controlled trial of a probiotic, Vsl#3, on gut transit and symptoms in diarrhoea-predominant irritable bowel syndrome. *Aliment Pharmacol Ther.* (2003) 17:895–904. 10.1046/j.1365-2036.2003.01543.x 12656692

[48] Kim HJ Vazquez Roque MI Camilleri M Stephens D Burton DD Baxter K A randomized controlled trial of a probiotic combination Vsl# 3 and placebo in irritable bowel syndrome with bloating. *Neurogastroenterol Motil.* (2005) 17:687–96. 10.1111/j.1365-2982.2005.00695.x 16185307

[49] Tap J Derrien M Tornblom H Brazeilles R Cools-Portier S Dore J Identification of an intestinal microbiota signature associated with severity of irritable bowel syndrome. *Gastroenterology.* (2017) 152:111–23e8. 10.1053/j.gastro.2016.09.049 27725146

[50] Simren M Barbara G Flint HJ Spiegel BM Spiller RC Vanner S Intestinal microbiota in functional bowel disorders: a rome foundation report. *Gut.* (2013) 62:159–76. 10.1136/gutjnl-2012-302167 22730468 PMC3551212

[51] Kassinen A Krogius-Kurikka L Makivuokko H Rinttila T Paulin L Corander J The fecal microbiota of irritable bowel syndrome patients differs significantly from that of healthy subjects. *Gastroenterology.* (2007) 133:24–33. 10.1053/j.gastro.2007.04.005 17631127

[52] O’Mahony L McCarthy J Kelly P Hurley G Luo F Chen K *Lactobacillus* and bifidobacterium in irritable bowel syndrome: symptom responses and relationship to cytokine profiles. *Gastroenterology.* (2005) 128:541–51. 10.1053/j.gastro.2004.11.050 15765388

[53] Verdu EF Bercik P Bergonzelli GE Huang XX Blennerhasset P Rochat F *Lactobacillus* paracasei normalizes muscle hypercontractility in a murine model of postinfective gut dysfunction. *Gastroenterology.* (2004) 127:826–37. 10.1053/j.gastro.2004.06.007 15362038

[54] Clos-Garcia M Andres-Marin N Fernandez-Eulate G Abecia L Lavin JL van Liempd S Gut microbiome and serum metabolome analyses identify molecular biomarkers and altered glutamate metabolism in fibromyalgia. *EBioMedicine.* (2019) 46:499–511. 10.1016/j.ebiom.2019.07.031 31327695 PMC6710987

[55] Minerbi A Gonzalez E Brereton NJB Anjarkouchian A Dewar K Fitzcharles MA Altered microbiome composition in individuals with fibromyalgia. *Pain.* (2019) 160:2589–602. 10.1097/j.pain.0000000000001640 31219947

[56] Vieira AT Macia L Galvao I Martins FS Canesso MC Amaral FA A role for gut microbiota and the metabolite-sensing receptor Gpr43 in a murine model of gout. *Arthritis Rheumatol.* (2015) 67:1646–56. 10.1002/art.39107 25914377

[57] Lanza M Filippone A Ardizzone A Casili G Paterniti I Esposito E Scfa treatment alleviates pathological signs of migraine and related intestinal alterations in a mouse model of Ntg-induced migraine. *Cells.* (2021) 10:2756. 10.3390/cells10102756 34685736 PMC8535085

[58] Bourdu S Dapoigny M Chapuy E Artigue F Vasson MP Dechelotte P Rectal instillation of butyrate provides a novel clinically relevant model of noninflammatory colonic hypersensitivity in rats. *Gastroenterology.* (2005) 128:1996–2008. 10.1053/j.gastro.2005.03.082 15940632

[59] Vanhoutvin SA Troost FJ Kilkens TO Lindsey PJ Hamer HM Jonkers DM The effects of butyrate enemas on visceral perception in healthy volunteers. *Neurogastroenterol Motil.* (2009) 21:952–e76. 10.1111/j.1365-2982.2009.01324.x 19460106

[60] Kannampalli P Shaker R Sengupta JN. Colonic butyrate– algesic or analgesic? *Neurogastroenterol Motil.* (2011) 23:975–9. 10.1111/j.1365-2982.2011.01775.x 21981302 PMC3191935

[61] Sharon G Sampson TR Geschwind DH Mazmanian SK. The central nervous system and the gut microbiome. *Cell.* (2016) 167:915–32. 10.1016/j.cell.2016.10.027 27814521 PMC5127403

[62] Du X Hao H Yang Y Huang S Wang C Gigout S Local gabaergic signaling within sensory ganglia controls peripheral nociceptive transmission. *J Clin Invest.* (2017) 127:1741–56. 10.1172/JCI86812 28375159 PMC5409786

[63] Cortes-Altamirano JL Olmos-Hernandez A Jaime HB Carrillo-Mora P Bandala C Reyes-Long S Review: 5-Ht1, 5-Ht2, 5-Ht3 and 5-Ht7 receptors and their role in the modulation of pain response in the central nervous system. *Curr Neuropharmacol.* (2018) 16:210–21. 10.2174/1570159X15666170911121027 28901281 PMC5883380

[64] Zhong S Zhou Z Liang Y Cheng X Li Y Teng W Targeting strategies for chemotherapy-induced peripheral neuropathy: does gut microbiota play a role? *Crit Rev Microbiol* (2019) 45:369–93. 10.1080/1040841X.2019.1608905 31106639

[65] Sm OM Dinan TG Cryan JF. The gut microbiota as a key regulator of visceral pain. *Pain.* (2017) 158(Suppl. 1):S19–28. 10.1097/j.pain.0000000000000779 27918315

[66] Cervero F Laird JM. Visceral pain. *Lancet.* (1999) 353:2145–8. 10.1016/S0140-6736(99)01306-9 10382712

[67] Brandt JS Hadaya O Schuster M Rosen T Sauer MV Ananth CV. A bibliometric analysis of top-cited journal articles in obstetrics and gynecology. *JAMA Netw Open.* (2019) 2:e1918007. 10.1001/jamanetworkopen.2019.18007 31860106 PMC6991228

