# Supplementary material for: Bibliometric and visual analysis of research on the links between the gut microbiota and pain from 2002 to 2021
Source: Front Med (Lausanne). 2022 Nov 15;9:975376. doi: 10.3389/fmed.2022.975376 (PMC9705792; doi:10.3389/fmed.2022.975376)
Supplement: Supplementary file 1 [file Data_Sheet_1.docx]

Supplementary Material


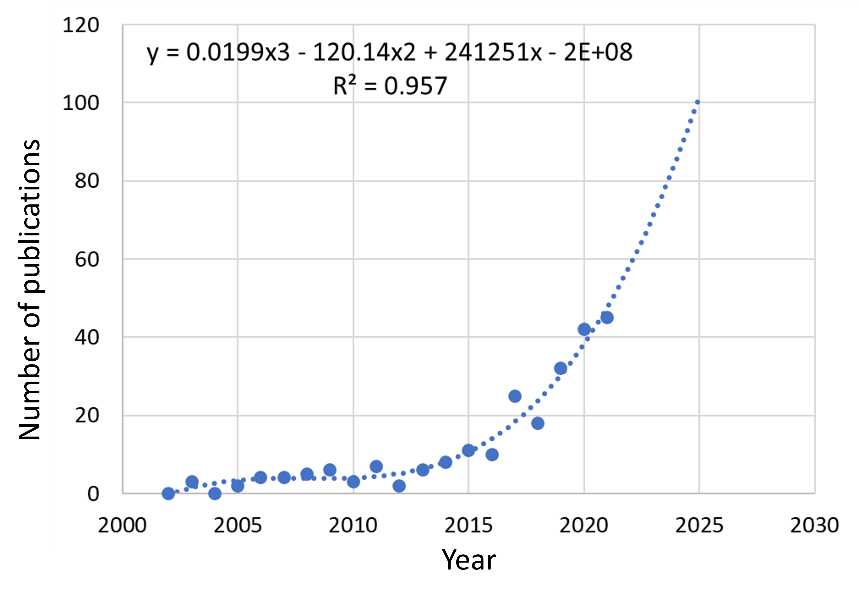


**Supplementary Figure 1**. Output of publications and growth prediction of research on the links between the gut microbiota and pain. The number of publications from 2002 to 2021 are presented by scatters. The dashed line represents the predicted curve, R^2^=0.957.

| **Types of pain** | | | **Number of articles** |
| --- | --- | --- | --- |
| Chronic pain | | |  |
|  | Chronic primary pain | |  |
|  |  | Chronic primary visceral pain - Irritable bowel syndrome | 67 |
|  |  | - Functional bowel disorders (except IBS) | 2 |
|  |  | - Chronic primary pelvic pain syndrome | 2 |
|  |  | - Chronic primary bladder pain syndrome | 1 |
|  |  | - Functional abdominal pain | 4 |
|  |  | Chronic widespread pain - Fibromyalgia syndrome | 4 |
|  |  | Complex regional pain syndrome | 1 |
|  |  | Chronic migraine | 6 |
|  |  | Chronic primary temporomandibular disorder pains | 1 |
|  | Chronic cancer related pain | |  |
|  |  | Chronic post cancer treatment pain | 7 |
|  |  | Chronic cancer pain | 1 |
|  | Chronic secondary visceral pain | | 9 |
|  | Chronic secondary musculoskeletal pain | |  |
|  |  | Chronic secondary musculoskeletal pain from persistent inflammation due to crystal deposition (gout) | 2 |
|  |  | Chronic secondary musculoskeletal pain from persistent inflammation due to autoimmune and auto-inflammatory disorders (PsA, RA) | 10 |
|  |  | Chronic secondary musculoskeletal pain associated with structural changes (lumbar disc herniation, OA) | 1 |
|  | Chronic neuropathic pain | | 11 |
|  | Chronic postsurgical or post traumatic pain | | 2 |
| Acute pain | | |  |
|  | Acute postoperative pain | | 4 |
|  | Others | | 1 |
| Others | | |  |
|  | Chronic low back pain (primary or secondary) | | 2 |
|  | Gulf War Illness | | 3 |
|  | Lyme disease | | 1 |
|  | Autism Spectrum Disorder | | 3 |
|  | Congenital insensitivity to pain with anhidrosis | | 1 |
|  | Chronic fatigue syndrome | | 1 |
|  | Menopausal symptoms | | 1 |
|  | Pain physiology | | 15 |
|  | Visceral pain and visceral sensitivity in the abdominal region | | 58 |
|  | Inflammatory pain | | 5 |
|  | Morphine treatment-related (analgesic tolerance, bacterial translocation) | | 7 |

**Supplementary Table 1**. The types of pain in the 233 articles. The classification was mostly based on ICD-11.
